# The Menace of the Bathing Pool

**Published:** 1912-09

**Authors:** 


THE MENACE OF THE BATHING POOL.—"As ordinarily run, swimming pools are little more than cesspools and clear themselves of bacteria largely on the septic tank principle," says H. F. J. Porter in the Survey.

"Investigations of public pools for a long period of time at Hamburg, as well as more recently of college pools at Purdue, Brown, Chicago and Yale universities, and the New York public swimming pools, shows that the impurities produced by bathers are bacterial and chemical foreign matter.

"Tests made at the Hamburg pools showed water fresh from the tap contained 57 microbes per cubic centimeter; the same volume after seventy-four persons had bathed contained 1800; after 494 persons 64,400 microbes were found in the tank. When tests were taken after 829 had bathed, however, only 154,000 microbes were found, illustrating the septic tank principle, that after a certain point has been reached the septic condition of the water either actually kills the microbes or else they devour each other until the excess is destroyed and an equilibrium representing the maximum impurity that the water can sustain is reached. The very expression of this condition which actually existed in all pools studied, is, it would seem, calculated to arouse alarm as well as disgust.

"In all pools some precautions can be used. Previous bathing and the exclusion of persons known to have contagious skin affections are the first, and these are enforced in few even of the large college pools. Frequent change of water is another essential.

"Where water is scarce other methods of purification can partly take the place of change, but there is none which can effectively and permanently keep up with an accumulation of bacteria due to many bathers in the same water.

"The quickest and cheapest method of purification, which is used frequently for purifying water supplies and which is applicable, it would seem, to some extent to river bathing places as well as indoor pools, is hypochloride of calcium, 1 part to 2,000,000 parts water, 20 pounds to 1,000,000 gallons of water."

